# Complete response to combination therapy using nivolumab and ipilimumab for metastatic, sarcomatoid collecting duct carcinoma presenting with high expression of programmed death-ligand 1: a case report

**DOI:** 10.1186/s13256-022-03426-3

**Published:** 2022-05-18

**Authors:** Takayoshi Fuu, Kazuyoshi Iijima, Yukiko Kusama, Toshiaki Otsuki, Haruaki Kato

**Affiliations:** 1 Department of Urology, Nagano Municipal Hospital, 1333-1, Tomitake, Nagano, Nagano Japan; 2 Department of Pathology, Nagano Municipal Hospital, 1333-1, Tomitake, Nagano, Nagano Japan; 3 Department of Pathology, Shinshu University Hospital, 3-1-1, Asahi, Matsumoto, Nagano Japan; 4 Department of Urology, Nagano Municipal Hospital, 1333-1, Oazatomitake, Nagano, Japan

**Keywords:** Collecting duct carcinoma, Sarcomatoid renal cell carcinoma, Immune checkpoint inhibitors, Programmed death-ligand 1, Immune-related adverse events, Case report

## Abstract

**Background:**

Collecting duct carcinoma and sarcomatoid renal cell carcinoma are tumors with poor prognosis. Immune checkpoint inhibitors have been established as the standard treatment for advanced renal cell carcinoma. Some cases of remission of collecting duct carcinoma and sarcomatoid renal cell carcinoma have been reported using immune checkpoint inhibitor interventions. Specifically, sarcomatoid renal cell carcinoma expresses high levels of programmed death-ligand 1, an immune checkpoint protein, and immune checkpoint inhibitors have been reported to be highly effective for treating sarcomatoid renal cell carcinoma.

**Case presentation:**

We describe the case of a 70-year-old Japanese male who underwent radical right nephrectomy for a right renal mass identified on computed tomography. The pathological examination demonstrated that the renal mass was urothelial carcinoma and collecting duct carcinoma with sarcomatoid changes, and programmed death-ligand 1 was highly expressed with a tumor proportion score of more than 10%. There was no evident submucosal connective tissue invasion in the urothelial carcinoma component, and collecting duct carcinoma was diagnosed as primary cancer. The tumor–node–metastasis classification was pT3aN0, venous invasion 1, lymphovascular invasion 0, and Fuhrman nuclear grade 4. Two months after the nephrectomy, multiple metastases were observed in both lungs, the right hilar lymph node, and the S6 segment of the right liver lobe. We initiated first-line combination therapy with nivolumab (240 mg, fixed dose) and ipilimumab (1 mg/kg). One day after administration, the patient developed drug-induced interstitial pneumonia, thus we applied steroid injections. After one administration of immunotherapy, the metastatic lesion showed complete response within 6 months, which was maintained after 3 years.

**Conclusion:**

We report the first case of complete response to a single dose of combination therapy with nivolumab and ipilimumab for metastatic collecting duct carcinoma with sarcomatoid changes and high expression of programmed death-ligand 1. This case suggests high expectations for immune checkpoint inhibitors as treatment for sarcomatoid-transformed renal carcinoma tumors that express high levels of programmed death-ligand 1.

## Background

Collecting duct carcinoma (CDC), also known as Bellini duct carcinoma, is a subtype of renal cell carcinoma (RCC) that develops in the distal collecting ducts of the kidney. CDC accounts for less than 2% of all RCCs [1] but is highly malignant. Metastasis is observed in > 70% of cases at diagnosis, and these patients display poor responses to chemotherapy and molecular targeted drugs with mean overall survival (OS) of approximately 1 year [2]. CDC with sarcomatous changes is even more infrequent. A PubMed search revealed only two case reports of sarcomatoid CDC [3, 4]. Nevertheless, the response to chemotherapy and radiotherapy is poor, and radical resection is the mainstay of treatment. Prognosis remains poor after resection.

Immune checkpoint inhibitors (ICIs) target molecules in the immune checkpoint pathway, such as programmed death 1 (PD-1), programmed death-ligand 1 (PD-L1), and cytotoxic T lymphocyte associated protein 4. PD-L1 is a cell surface protein that binds to PD-1 on activated T lymphocytes and reduces their antitumor activity [5]. ICIs have recently been introduced, and combined ICI therapy has become the standard treatment for advanced clear cell renal cell carcinoma (CCRCC). In the CheckMate 214 phase III trial, combined treatment with ICIs nivolumab and ipilimumab significantly prolonged OS compared with sunitinib alone in untreated, advanced CCRCC patients who were categorized as at intermediate or poor risk by the International Metastatic RCC Database Consortium (IMDC) risk score [6].

Sarcomatoid RCC (sRCC) accounts for 4–5% of RCCs, and nearly 20% of metastatic RCCs are accompanied by sarcomatoid changes [7]. The postoperative recurrence rate of sRCC is higher, and the survival rate is lower at any disease stage than that of non-sRCC [8, 9]. In recent years, the application of ICIs has expanded, and the relationship between the expression of PD-L1 and therapeutic efficacy of ICIs has attracted substantial attention. High levels of PD-L1 expression are observed in sRCC compared with other RCCs, and ICIs are potentially effective against sRCC [10]. A recent report demonstrated that nivolumab and ipilimumab improved OS, progression-free survival (PFS), and complete response (CR) rates compared with sunitinib in a post hoc analysis of sRCC patients from the CheckMate 214 study [11].

## Case presentation

**Fig. 1:**
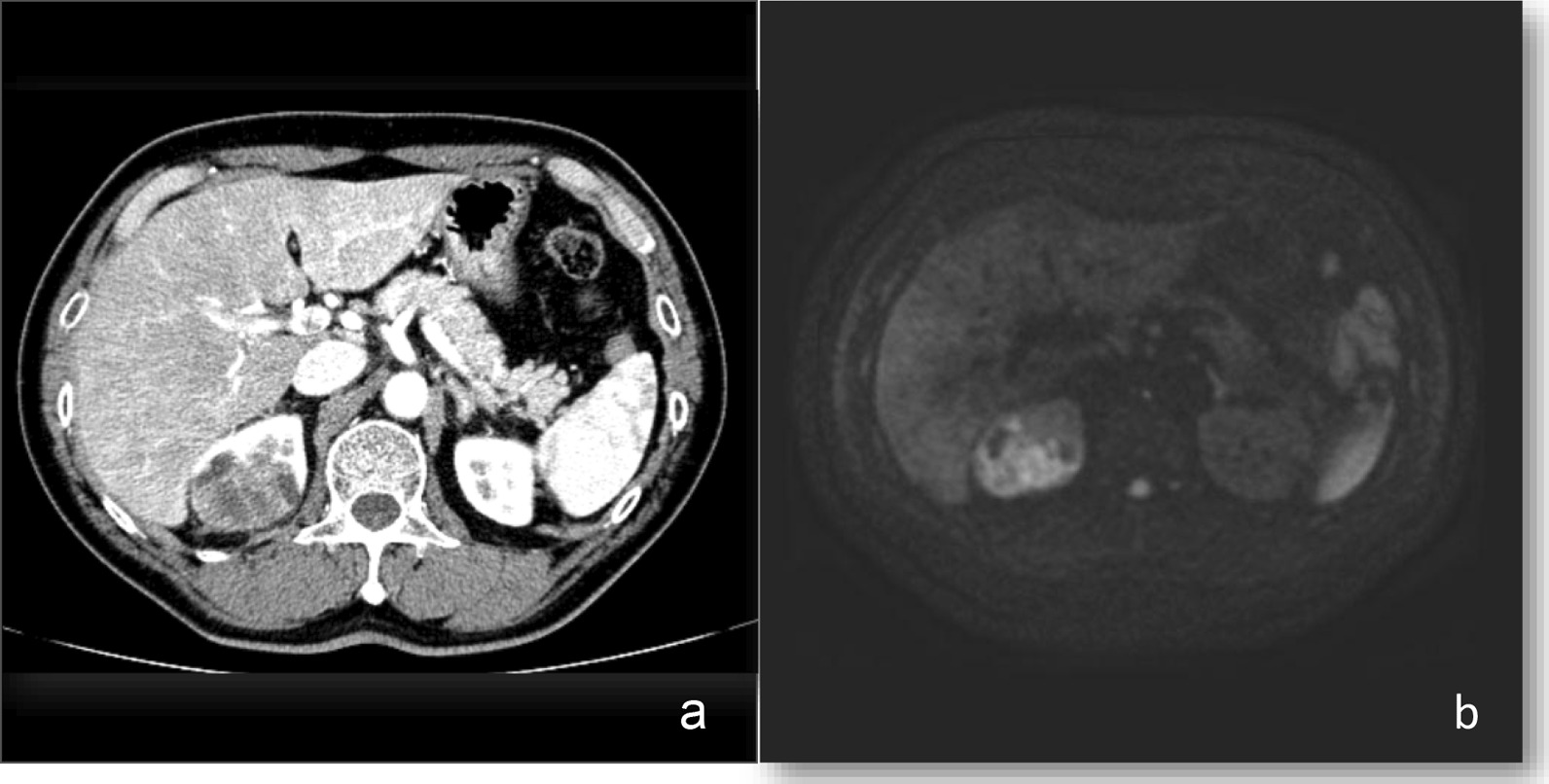
Abdominal imaging of the patient at diagnosis. **a** Contrast-enhanced, early-phase CT scan revealed an internal heterogeneous mass with poor contrast **b** diffusion-weighted magnetic resonance imaging showed a high signal intensity

**Fig. 2:**
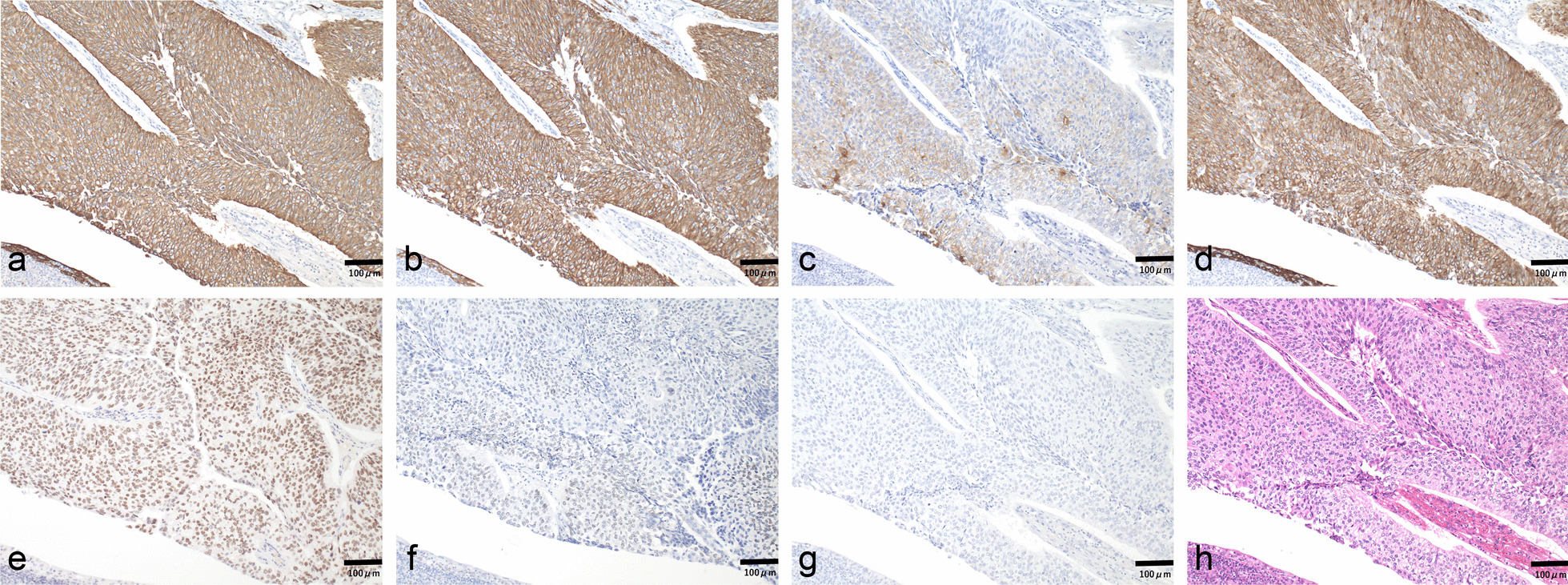
Immunostaining of urothelial carcinoma from the nephrectomy specimens (original magnification ×100). Tissue sections were stained with antibodies against **a** CK-AE1/AE3, **b** CK7, **c** CK20, **d** CK-34β1E12, **e** GATA3, **f** p63, and **g** PAX8. Brown coloring indicates positive antibody staining. **h** Hematoxylin and eosin-stained sections

**Fig. 3:**
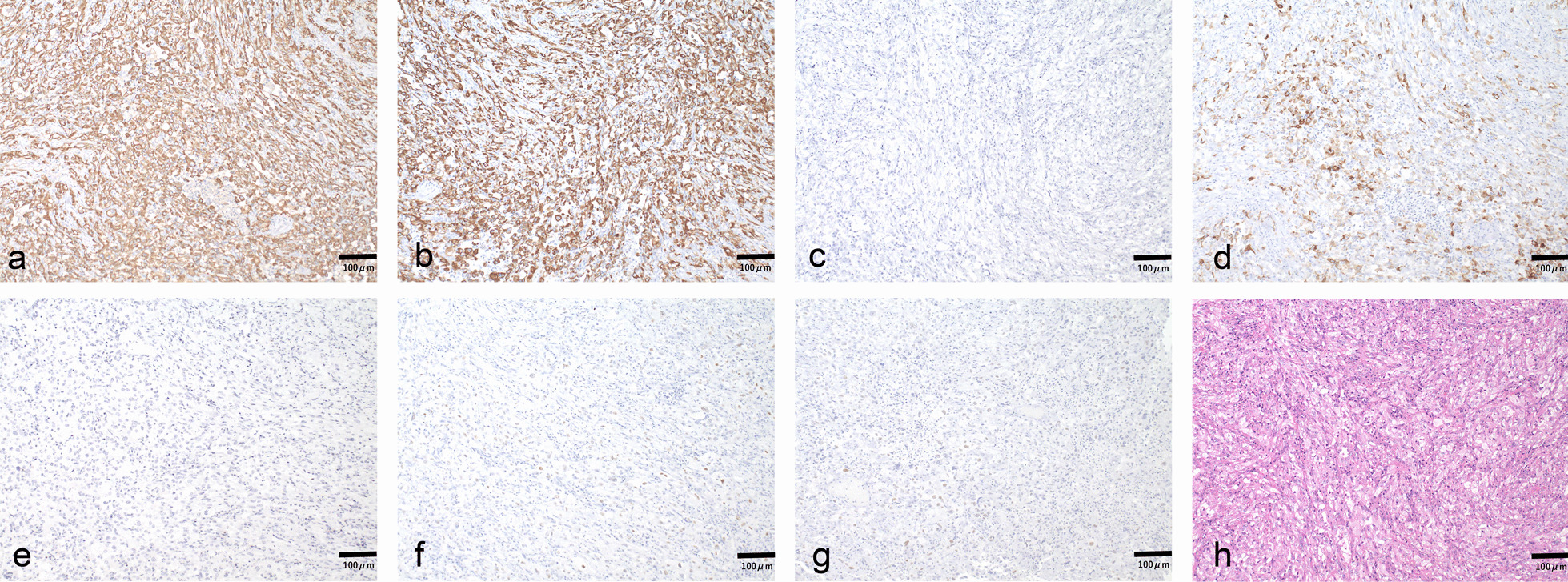
Immunostaining of collecting duct carcinoma components from the nephrectomy specimens (original magnification ×100). Tissue sections were stained with antibodies against **a** CK-AE1/AE3, **b** CK7, **c** CK20, **d** CK-34β1E12, **e** GATA3, **f** p63, and **g** PAX8. Brown coloring indicates positive antibody staining. **h** Hematoxylin and eosin-stained sections

**Fig. 4:**
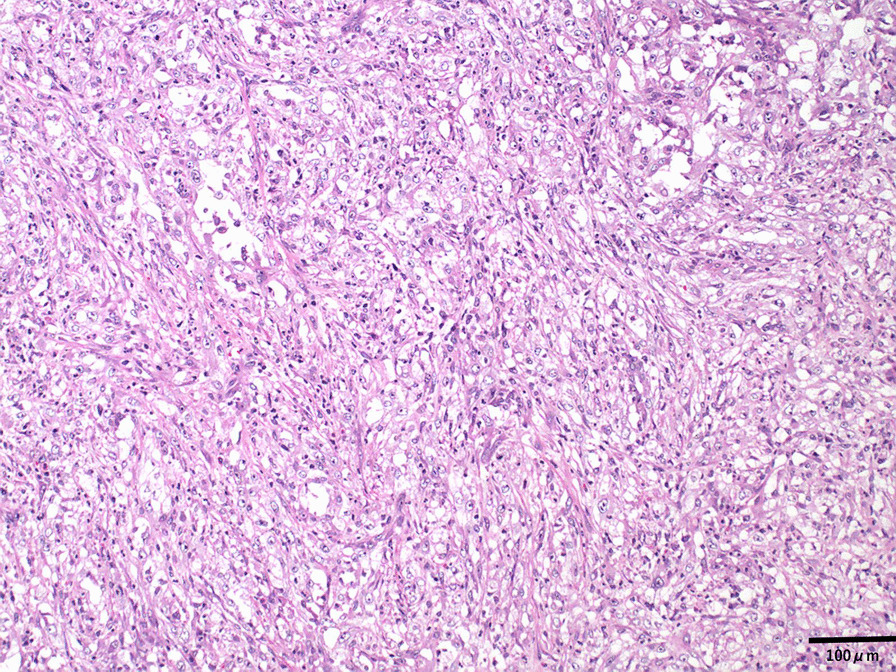
Hematoxylin and eosin staining of collecting duct carcinoma with sarcomatoid changes. Tissue sections were prepared from the nephrectomy specimens (original magnification ×100)

**Fig. 5:**
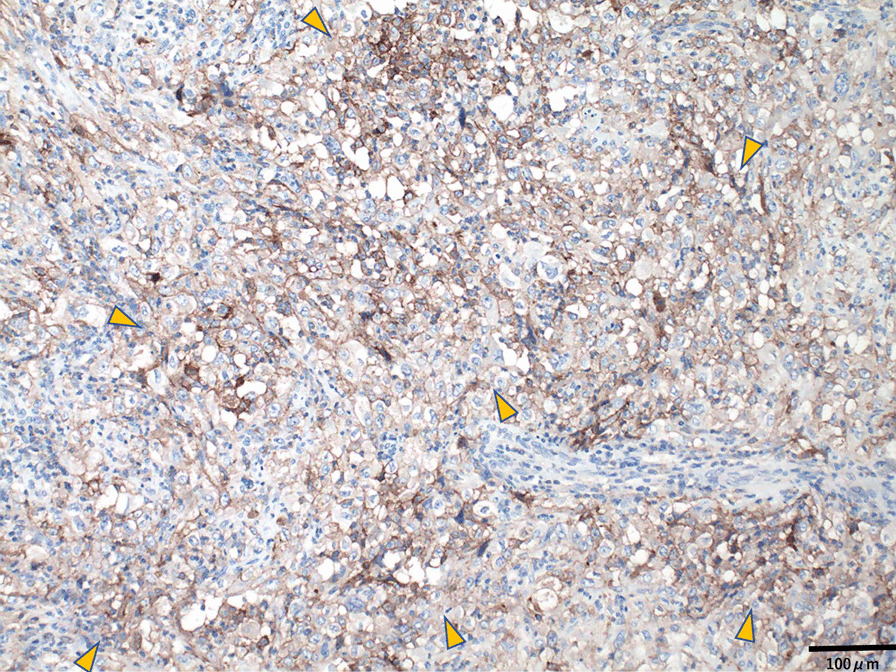
Expression of programmed death-ligand 1 in sarcomatoid tissue from nephrectomy specimens (original magnification ×100). Brown coloring indicates positive antibody staining (yellow arrows), and the tumor proportion score is more than 10%

A 70-year-old Japanese male was referred to our department for a diffusely spreading mass in the upper right pole of the renal parenchyma, which was observed on follow-up computed tomography (CT) scan 1 year after endoscopic submucosal dissection for rectal cancer. At the initial examination, there were no abnormalities in physical and laboratory findings. Contrast-enhanced, early-phase CT scan revealed an internal heterogeneous mass with poor contrast, and diffusion-weighted magnetic resonance imaging showed a high signal intensity (Fig. 1). However, no obvious metastases were observed. Percutaneous needle biopsy indicated the presence of poorly differentiated carcinoma, ductal carcinoma in situ, and spindle cell carcinoma in situ with sarcomatous changes. Therefore, a radical right nephrectomy was performed. Pathological examination of the kidney revealed the presence of CDC with sarcomatoid features and urothelial carcinoma. The gross findings demonstrated cysts containing small stones in part, and the urothelial carcinoma component in that area. The uroepithelial components were cytokeratin (CK)-AE1/AE3(+); CK7(+); CK20(+), partial; CK-34β1E12(+); GATA3(+); p63(+), partial and weak; and PAX8(−) (Fig. 2). The CDC components were CK-AE1/AE3(+); CK7(+); CK20(−); CK-34β1E12(+), partial; GATA3(−); p63(+), partial and weak; and PAX8(+), partial (Fig. 3). Spindle-shaped cells as well as hobnail patterns and carcinoma in situ were observed in the CDC tissue (Fig. 4), and immunohistochemistry using an anti-PD-L1 (clone 28-8) antibody demonstrated that PD-L1 was highly expressed with a tumor proportion score of more than 10% (Fig. 5). Despite the diagnosis of double cancer, we did not observe evident submucosal connective tissue invasion from the urothelial carcinoma component and therefore concluded that CDC was the primary cancer. The TNM Classification of Malignant Tumors (UICC7th) for this case was pT3aN0, venous invasion 1, lymphovascular invasion 0, Fuhrman nuclear grade 4.

**Fig. 6:**
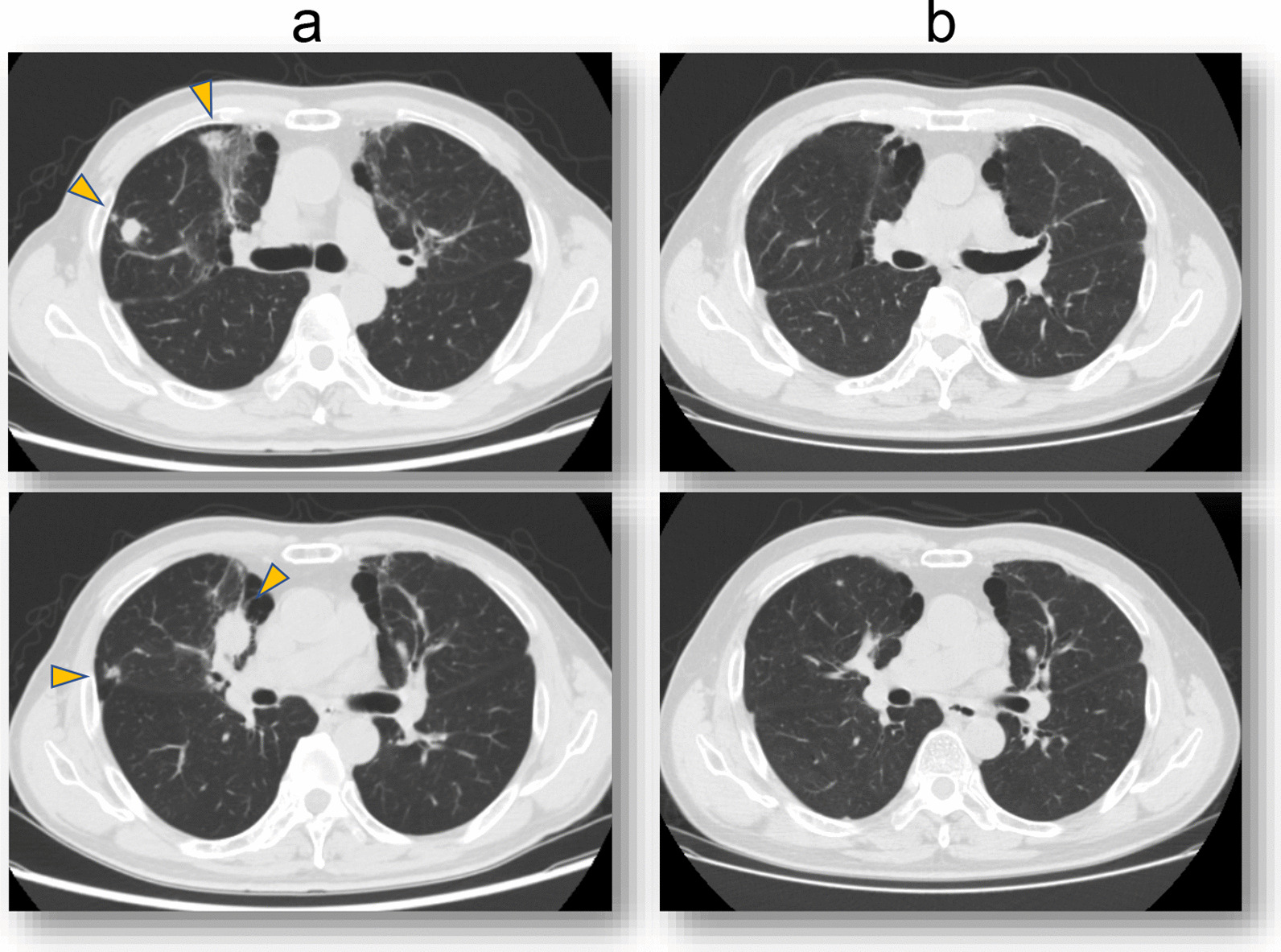
Metastatic lesions (yellow arrows) **a** immediately and **b** at 20 months after the administration of nivolumab and ipilimumab

Two months after the surgery, a follow-up CT scan showed multiple metastases in both lungs, right hilar lymph node, and the S6 segment of the right liver lobe. Nivolumab and ipilimumab combination therapy (fixed dose of 240 mg and 1 mg/kg body weight, respectively) was initiated in the third postoperative month. Fever was observed 1 day after ICI administration, and CT scan performed 3 days later showed interstitial pneumonia. Bronchoscopy was not performed, but after consultation with a pulmonologist, a diagnosis of drug-induced interstitial pneumonia was made and methylprednisolone (4 mg/kg/day) was administered. No further tumor treatment was provided after the initial administration of ICIs. However, the metastatic lesion showed CR to treatment within 6 months, and the CR was maintained after 3 years as evidenced by follow-up CT scans every 2 months (Fig. 6). Interstitial pneumonia was treated with a gradual decrease in steroid dosage and terminated after 500 days; no flare-ups of pneumonia were observed on CT scans every 2 months for 3 years.

## Discussion and conclusions

CDC is an infrequent RCC subtype with poor prognosis, and the majority of patients with CDC die within 3 years of diagnosis [1]. Although the local treatment is radical nephrectomy, there is no established systemic therapy for advanced CDC [2]. The CheckMate 214 trial demonstrated that ICIs significantly improved OS compared with conventional therapies for advanced RCC. Combined therapy with nivolumab and ipilimumab has become the first-line treatment for RCC patients classified as at intermediate or poor risk by the IMDC risk score system [6]. Although data for CDC are limited, there have been several reports describing positive CDC patient responses to ICI treatment, including CR cases [12–16]. Two previous cases suggested an association between high PD-L1 expression in tumor tissues and therapeutic efficacy of ICIs [12, 13]. Additionally, in the previous two reports, combination immunotherapy with nivolumab and ipilimumab was used against metastatic CDC [14, 16]. Malouf *et al.* also reported activation of the immune system in CDC, including high levels of tumor-infiltrating CD3^+^ and CD8^+^ lymphocytes, suggesting that treatment response to ICIs is readily attained [14, 17]. Given these findings, despite the absence of data on CDC, combined treatment with nivolumab and ipilimumab was introduced for this patient.

The sarcomatoid subtype of RCC has poor prognosis, and there is no established treatment for advanced sRCC [7–9]. With the expanded application of ICIs, the relationship between PD-L1 expression and therapeutic efficacy of ICIs has been demonstrated. PD-L1 expression in RCC has conventionally been associated with markers of poor prognosis, such as increased International Society of Urological Pathology grade, necrosis, and sarcomatoid changes [18]. A recent metaanalysis of ICI treatment for RCC reported a higher therapeutic response rate to ICIs in sRCC patients compared with that observed for non-sRCC patients, suggesting an association between high expression of PD-L1 in sRCC and high therapeutic efficacy of ICIs [10, 19].

This case demonstrated that a tumor with sarcomatoid changes, which is a subtype of CDC with poor prognosis, showed CR after combinatorial ICI treatment. Although CDC with sarcomatoid changes is rare, the fact that sarcomatoid changes were observed may be a factor that facilitated the therapeutic effect of the ICIs because the PD-L1 expression level in the sarcomatoid tissue of this specimen was elevated. As in other RCC subtypes, CDC with sarcomatoid changes in this case supported a high therapeutic efficacy of the ICIs.

An immune-related adverse event (irAE) was observed 1 day after ICI injections in this case. Previous studies reported a relationship between irAEs and the antitumor effect of ICIs in malignant melanoma and non-small cell lung cancer, resulting in significant prolongation of OS [20, 21]. A similar report has been published for RCC [22]. This case may also suggest an association between an irAE and complete response to nivolumab and ipilimumab.

Recent experimental studies have hinted at a role for mitochondria in determining the response to anti-PD-1 immunotherapy [23]. Authors mentioned that male patients with older age have a higher chance of complete response to anti-PD-1 immunotherapy through less potent mitochondria or mitochondrial abnormalities and increased PD-1 expression on T cells. In this case report, better early response rate was in the form of complete response and translated to prolonged disease-free survival.

This case showed distant metastasis 2 months after surgery. A recent paper posited that cancer cells already present in the body are in a quiescent stage of the cell cycle in order to survive in a harsh environment [24]. According to this hypothesis, metastatic recurrence of a tumor occurs after surgical removal of the primary tumor, when nutrients are transferred to the quiescent cancer cells and they restart proliferation.

This is the first report of a patient who achieved CR with a single dose of nivolumab and ipilimumab combination therapy for sarcomatoid CDC with multiple lung, lymph node, and liver metastases 2 months post-nephrectomy. The standard therapy for metastatic CDC is chemotherapy or tyrosine kinase inhibitors. However, precedent cases, including this one, suggest that treatment with ICIs may be a better option than conventional chemotherapy or molecular targeted therapy. In the presence of sarcomatoid tumor changes and high expression of PD-L1, therapy with ICIs for metastatic CDC might be considered as a first-line treatment based on this and other case reports, and this therapy may also be more effective in the absence of chemotherapy. To establish systemic treatment regimens for advanced CDC, further studies with indicators, such as the presence of sarcomatoid changes, its associated biomarker expression, and irAEs, are needed.

In conclusion, we observed the first case of complete response to a single dose of combination therapy using nivolumab and ipilimumab for metastatic, sarcomatoid CDC with high expression of PD-L1. This case supports high expectations for ICIs for sarcomatoid-transformed renal cell carcinoma that expresses high levels of PD-L1.

## Data Availability

Not applicable.

## Abbreviations

CCRCC: Clear cell renal cell carcinoma
CDC: Collecting duct carcinoma
CR: Complete response
CT: Computed tomography
ICIs: Immune checkpoint inhibitors
IMDC: International Metastatic RCC Database Consortium
irAE: Immune-related adverse events
OS: Overall survival
PD-1: Programmed death 1
PD-L1: Programmed death-ligand 1
PFS: Progression-free survival
RCC: Renal cell carcinoma
sRCC: Sarcomatoid renal cell carcinoma
